# Rat1 promotes premature transcription termination at R-loops

**DOI:** 10.1093/nar/gkae033

**Published:** 2024-01-28

**Authors:** José Antonio Mérida-Cerro, Pablo Maraver-Cárdenas, Ana G Rondón, Andrés Aguilera

**Affiliations:** Centro Andaluz de Biología Molecular y Medicina Regenerativa-CABIMER, Universidad de Sevilla, CSIC, 41092 Seville, Spain; Departamento de Genética, Facultad de Biología, Universidad de Sevilla, 41012 Seville, Spain; Centro Andaluz de Biología Molecular y Medicina Regenerativa-CABIMER, Universidad de Sevilla, CSIC, 41092 Seville, Spain; Departamento de Genética, Facultad de Biología, Universidad de Sevilla, 41012 Seville, Spain; Centro Andaluz de Biología Molecular y Medicina Regenerativa-CABIMER, Universidad de Sevilla, CSIC, 41092 Seville, Spain; Departamento de Genética, Facultad de Biología, Universidad de Sevilla, 41012 Seville, Spain; Centro Andaluz de Biología Molecular y Medicina Regenerativa-CABIMER, Universidad de Sevilla, CSIC, 41092 Seville, Spain; Departamento de Genética, Facultad de Biología, Universidad de Sevilla, 41012 Seville, Spain

## Abstract

Certain DNA sequences can adopt a non-B form in the genome that interfere with DNA-templated processes, including transcription. Among the sequences that are intrinsically difficult to transcribe are those that tend to form R-loops, three-stranded nucleic acid structures formed by a DNA-RNA hybrid and the displaced ssDNA. Here we compared the transcription of an endogenous gene with and without an R-loop-forming sequence inserted. We show that, in agreement with previous *in vivo* and *in vitro* analyses, transcription elongation is delayed by R-loops in yeast. Importantly, we demonstrate that the Rat1 transcription terminator factor facilitates transcription throughout such structures by inducing premature termination of arrested RNAPIIs. We propose that RNase H degrades the RNA moiety of the hybrid, providing an entry site for Rat1. Thus, we have uncovered an unanticipated function of Rat1 as a transcription restoring factor opening up the possibility that it may also promote transcription through other genomic DNA structures intrinsically difficult to transcribe. If R-loop-mediated transcriptional stress is not relieved by Rat1, it will cause genomic instability, probably through the increase of transcription-replication conflicts, a deleterious situation that could lead to cancer.

## Graphical Abstract

**Graphical Abstract:**
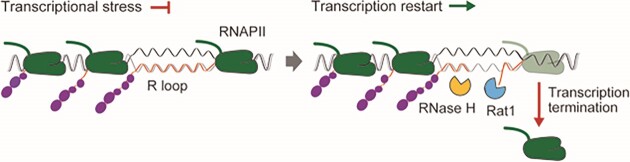

## Introduction

Transcription elongation is a discontinuous process with RNA polymerases (RNAP) transiently pausing or even stalling at different sites, as promoter proximal regions, natural barriers formed by specific chromatin disposition, DNA damage sites or highly structured DNA sequences (1). Under physiological conditions, most of the genome is in the canonical B-form, a right-handed double helix. However, studies based on antibodies that specifically recognize alternative DNA structures have demonstrated the existence of other non-canonical DNA forms in living cells: G-quadruplexes, Z-DNA, cruciform structures, harpins and R-loops, among others (2–4). These structures could play a physiological role in the cell by regulating gene expression, but they could also impose a physical barrier to DNA-templated processes such as replication or transcription, and could subsequently cause genome instability (5).

R-loops are non-canonical nucleic acid structures formed by a DNA–RNA hybrid and the displaced single-stranded DNA (ssDNA), which can contain G-quadruplexes or be coated by ssDNA binding proteins: RPA or AtNDX (6–8). R-loops may have a variety of positive functions, as in transcription regulation or immunoglobulin variability. However, unscheduled R-loops lead to genome instability (9–11). As a result, cells have evolved several mechanisms to preserve their genomes by limiting R-loops throughout the different phases of the cell cycle, either by resolving or preventing their formation (12). R-loop resolvases are RNase H and several RNA–DNA helicases, which degrade or unwind the RNA from the hybrid, respectively (11,13). The activities that prevent R-loop accumulation are DNA topoisomerases or proteins involved in co-transcriptional assembly of the nascent RNA into a mature messenger ribonucleoprotein (mRNP). Both processes prevent RNA from threading back into template DNA, limiting DNA melting or RNA accessibility respectively (10,11). The THO complex was the first mRNP assembly factor identified to prevent R-loop formation (14). The yeast THO complex is a heteropentameric complex formed by Hpr1, Tho2, Mft1, Thp2 and Tex1, and it is conserved in higher eukaryotes with three additional subunits (THOC4, THOC5 and THOC6) (15). Although several other RNA-binding factors prevent R-loop formation presumably by coating the nascent RNA (13,16), the THO complex may also recruit the DNA–RNA helicase Sub2/UAP56, recently identified as a major activity limiting R-loops during transcription (17).

R-loops affect transcription differently depending on their location. At promoters, R-loops can either prevent initiation by blocking RNAPII access or facilitate it by opening the chromatin (8,18–21). In the 3′-regions of mammalian genes, they can promote transcription termination, possibly by pausing RNAPII through heterochromatin formation (22), whereas in the body of the genes, R-loops interfere with transcription elongation. Thus, *in vitro*, a DNA-RNA hybrid reduces the efficiency of transcription elongation, and *in vivo* mutants that accumulate R-loops, such as THO mutants, show a transcription elongation defect (23–27). However, it is not clear whether the DNA-RNA hybrid acts as a physical barrier to incoming RNA polymerases or whether the RNAP producing the nascent RNA that forms the hybrid is the one blocked. In support of the first model, *in vitro* RNAPII elongation through a template with a preformed R-loop is reduced (28,29). On the other hand, if an elongating RNAPII is attached to the DNA by its RNA, the helical nature of DNA could cause wrapping of the nascent RNA around it, increasing the probability of RNAPII pausing or blocking (30). The reverse situation may also happen with transcriptional stress leading to R-loop formation (31). This, together with the facts that transcription and R-loops interfere with DNA replication causing genome instability, support the current view that cells must have specific mechanisms to counteract the negative impact of R-loops on transcription.

To explore how cells respond to RNAPII transcription impairment caused by R-loops, we inserted an R-loop-prone sequence with a high GC skew, obtained from the murine immunoglobulin heavy chain μ switch region (Sμ350), into the endogenous *LYS2* gene of *Saccharomyces cerevisiae*. We first showed that the R-loop-prone sequence reduces RNAPII transcription elongation. Interestingly, we found that the Rat1 transcription factor promotes premature transcription termination of stalled RNAPIIs, thus eliminating the transcriptional stress caused by R-loops. Consistently, depleting Rat1 from the cell increased DNA damage in an RNase H-sensitive manner. Thus, our results support the view that unscheduled R-loops are harmful to cells by impairing replication and also transcription. Importantly, they also provide a mechanism by which cells counteract the negative impact of R-loops on transcription by promoting premature transcription termination via Rat1. Our study raises the possibility that this mechanism may also facilitate transcription through other non-canonical DNA structures.

## Materials and methods

### Yeast strains, plasmids and media

Yeast strains used in this study are derivatives of W303 (*MATa his3-11,15 leu2-3 112 Δtrp1 ura3-1, ade2-1 can1-100*). They are listed in Supplementary Table S1. The GLY strain, in which the *LYS2* promoter on chromosome II was replaced by the *GAL1* promoter from pFA6-NATnt2-GALp (32), was previously described (33). The GLSd strain containing the murine Sμ350 fragment in the position 2952 of the *LYS2* gene was obtained by transforming GLY strain with a PCR cassette amplified from pRS413-SF (34) using ‘Sm350 LYS2 fw’ and ‘rv’ primers (Supplementary Table S3) and pML104-LYS2A (33), a vector expressing Cas9 and a guide RNA that target position 2952 of *LYS2* gene. GLY-B and GLSd-B Mat alpha strains were obtained by genetic crosses with YBP250; GLYH and GLSdH were obtained by crossing GLY-B or GLSd-B with HPBAR1-R (12) and GLYRH and GLSdRH by disrupting *RNH1* and *RNH201* genes in GLY and GLSd with PCR cassettes obtained from pFA6a-kanMX6 (35) using primers ‘RNH1-MX6 fw’ and ‘rv’ or from pFA6a–hphNT1 (32) using primers ‘RNH201-Hyg fw’ and ‘rv’, respectively.

RATDG and SPTDG strains were obtained by insertion of a *AID::9myc::hphNT1* PCR cassette obtained from pHyg-AID-9myc (36) using primers ‘RAT1-AID fw’ and ‘rv’, or ‘SPT5-AID fw’ and ‘rv’, respectively, in the C-terminal end of *RAT1* and *SPT5* genes of the YMK612 strain (37). GLYRT, GLSdRT, GLYSPT and GLSdSPT were obtained by genetic crossing between GLY-B and GLSd-B with RATDG and SPTDG. GLYDF and GLSdDF were obtained by tagging the *DEF1* gene with *AID::9myc::hphNT1* PCR cassette from pHyg-AID-9myc using primers ‘DEF1-AID fw’ and ‘rv’ in GLYDG and GLSdDG respectively.

The generation of the YLYS2 strain, containing a single bp deletion at position 3705 of the *LYS2* gene, was previously described (33). The allele *rat1-1* (RAT1-Y657C) was introduced by CRISPR-cas9 in YLYS2 (strain YLR2) and in GLSd (GLSR). DGLSdRT was obtained by mating YLR2 and GLSR rat1-1 strains. DGLSd was generated mating YLYS2 and GLSd.

Plasmids used in this study were described in previous studies and are listed in Supplementary Table S2.

Media used in this study: YPAD (1% yeast extract, 2% bacto-peptone, 2% glucose, and 20 mg/ml adenine), synthetic defined (SD) (0.17% yeast nitrogen base without amino acids, 0.5% ammonium sulphate, supplemented with amino acids), synthetic complete (SC) (SD with 2% glucose, 2% galactose or 2% raffinose). Solid media was prepared by adding 2% agar. For protein depletion, the degron-tagged strains were grown in media with 1 mM of auxin (1-naphthaleneacetic acid, NAA, from Sigma) for 120 min. Yeast strains were defrosted from glycerol stocks and grown at 30°C. Yeast and bacteria culture, cloning and PCR were performed following standard procedures.

### RNA isolation from yeast

RNA was extracted from mid-log cultures with acid phenol after 1 h of galactose induction following standard procedures. Briefly, mid-log cultures were collected and washed in cold water prior to resuspend the cell pellet in TES buffer (10mM Tris, 1mM EDTA, 0.5% SDS in DEPC treated water) and immediately mixed with acid phenol (Sigma). Samples were incubated at 65ºC during 45 minutes with eventual vortexing. Aqueous phase was recovered and RNA was precipitated with cold ethanol.

### Northern blot assay

RNA was separated by agarose electrophoresis in a gel containing MOPS 1× and 2% formaldehyde and transferred to Hybond-N nitrocellulose membranes (GE Healthcare). *LYS2* and *SCR1* probes were amplified by PCR using primers ‘LYS2 probe A fw’ and ‘rv’ or ‘SCR1 .483 rv’ and ‘SCR1 .99 dw’, respectively, purified using Macherey-Nagel's DNA extraction kit and ^32^P-labelled using Klenow polymerase (Roche) and ^32^P-dCTP. Signal was acquired using a FLA-5100 Imager Fluorescence Analyzer (Fujifilm) and quantified with MultiGauge 2.0 analysis software (Science Lab). Signal was measured as arbitrarily units (a.u.) and normalized to control condition.

### Western blot assay

Western blots were performed on proteins extracted with 10% TCA following standard procedures. Samples were loaded on acrylamide gels, migrated in an SDS-containing buffer, transferred to nitrocellulose membranes and hybridized with anti-myc primary antibody and anti-mouse HRP-conjugated secondary antibody to detect the degron-tagged protein and Tir1-myc in each strain before and after auxin treatment at the indicated dilutions (Supplementary Table S4). Images were acquired using Amersham ImageQuant 800 (Cytiva).

### Chromatin immunoprecipitation (ChIP)

For steady-state RNAPII ChIP, 50 ml of mid-log cultures were grown overnight in galactose SC, crosslinked with 1% formaldehyde and quenched with glycine. Chromatin was processed as previously described (38) with some modifications: Cells were resuspended in cold lysis buffer (50 mM HEPES–KOH at pH 7.5, 150 mM NaCl, 1 mM EDTA, 1% Triton X-100, 0.1% sodium deoxycholate, 0.1% SDS, 1 mM phenylmethanesulfonyl fluoride, EDTA-free protease inhibitor) and lysed with glass beads in a VXR basic (Vibrax) multivortex at 4ºC. Chromatin was then sonicated to an average fragment size of 300–500 bp using a Bioruptor UCD-200 (Diagenode) and immunoprecipitated with different antibodies (Supplementary Table S4) that were previously bound to Dynabeads Protein A or G (Invitrogen). 1/10 of the chromatin was used as input. Chromatin was eluted and DNA released by protein digestion during 2 h at 42ºC with 1 ng/μl Pronase (Sigma) and decrosslinked during 5 h at 65ºC. DNA was then purified using Macherey-Nagel purification kit. For kinetics after transcription shut down, overnight cultures in raffinose SC were supplemented with galactose (2% final concentration) during 2 h to induce transcription and glucose was added to 2% final concentration in order to inhibit transcription. Short time points were collected and immediately crosslinked with 1% formaldehyde.

### DNA–RNA hybrid immunoprecipitation (DRIP)

Transcription was induced in 100 ml mid-log cultures grown in raffinose SC medium by addition of 2% galactose 16 h before collecting the sample. DRIP was performed as previously described (39). Briefly, spheroplasts were made by using spheroplasting buffer (1 M sorbitol, 2 mM Tris pH 8.0, 100 mM EDTA pH 8.0, 0.1% ß-mercapto-ethanol) with 2 mg/ml Zymolyase 20T (USB). Spheroplasts were lysed in cold Solution I (0.8 mM GuHCl, 30 mM Tris pH 8.0, 30 mM EDTA pH 8.0, 5% Tween-20, 0.5% Triton X-100) and treated with RNase A (Roche) and then with proteinase K (Roche). Extracts were cleared with chloroform-isoamyl alcohol (24:1) and nucleic acids precipitated with isopropanol and spooled on a glass rod. After washing with 70% ethanol and dried, DNA pellets were resuspended in TE and digested with HindIII, EcoRI, XbaI, SspI and BsrGI restriction enzymes (New England BioLabs) overnight. Half of each sample was treated with 8 μl RNase H (New England BioLabs) overnight as negative control. 1/5 of each sample was processed as input and DNA-RNA hybrids were immunoprecipitated using S9.6 antibody bound to Dynabeads Protein A (Invitrogen), cleaned with binding buffer (10 mM NaPO_4_ pH 7.0, 140 mM NaCl, 0.05% Triton X-100) and eluted with elution buffer (50 mM Tris pH 8.0, 10 mM EDTA, 0.5% SDS) plus proteinase K. DNA was cleaned using Macherey-Nagel purification kit. The relative abundance of DNA-RNA hybrids in each region was normalized to their input signal and compared to RNase H treated samples to confirm DNA-RNA hybrid immunoprecipitation.

### Quantitative PCR

qPCR experiments in this work follow the recommended ‘MIQE Guidelines for qPCR'. qPCR primers were designed using Primer Express software (Applied Biosystems) with an amplicon size of 100–250 bp and Tm ≥ 58ºC. Their specificity was checked by BLAST in *Saccharomyces Genome Database* tools comparing each primer sequence with the W303 strain genome using the default BLOSUM62 comparison matrix and a cutoff score (*E* value) of 0.01 (Supplemental Table S3). Quantitative PCR were performed at given regions using SYBR Green PCR Master Mix (Biorad) in a 7500 Fast Real-Time PCR system (Applied Biosystems) following the manufacturer protocol. Relative quantification of the samples was perform referred to a standard curve and the melting curve for each primer set was analysed.

### Genome-wide data analysis

The dataset with accession [GSE79222] was used for the ChIP-seq analysis (40). Paired-end data was aligned using Bowtie2 (v2.5.0) with default settings (41). The generated SAM files were sorted using SAMtools (V1.9) (42). Duplicate reads were then removed using the RmDup function of SAMtools. Coverage analysis was performed with the bamCoverage function from deepTools (v3.5.2) (43), with a specified bin size of 1 and normalization method set to CPM (Counts Per Million). Two replicate bigwig files were produced from the coverage analysis. These were combined using the bigwigCompare function to produce a mean ChIP file plotted.

The dataset with accession [GSE159870] was used for the DRIPc-seq analysis (12). We used Wild Type G1-DRIPc-seq data. Reads were aligned to the *Saccharomyces cerevisiae* reference genome (sacCer3) using Rsubread (V2.0.1) software with the unique = TRUE option (44). The generated SAM files were sorted using SAMtools (V1.10) (42). Duplicate reads were then removed using the RmDup function of SAMtools. Reads were assigned to Watson or Crick strands with SAMtools. Coverage analysis was performed using the bamCoverage function from deepTools (v3.5.2) (43), with a specified bin size of 10 and normalization method set to RPKM. DRIPc-seq peak calling was performed using ChromstaR V1.12.0 with pre-set false discovery rate parameters (45).

Heatmaps were generated using the deeptools package (46), while Genome examples were plotted using IGV (v2.16.2) and sacCer3 features.

We utilized the public servers of usegalaxy.eu in conjunction with our in-house resources to execute all computational analyses previously described.

### Rad52 foci analysis

Spontaneous Rad52 foci formation was visualized in yeast containing the pWJ1344 plasmid that expresses the Rad52-YFP tagged protein. Cells were fixed with 2.5% formaldehyde and washed with phosphate buffer at pH 6.4, pH 6.6 and pH 7.4. Nuclei were stained with DAPI prior to microscopy. At least 200 S/G2 phase cells were analysed in each sample by fluorescent microscopy on a DM600B microscope (Leica), as previously described (47).

### Genetic analysis of recombination

For recombination assays, the indicated strains containing the *LYS2* diploid system were grown in galactose medium during 3–4 days. *rat1-1* mutants were cultured at the semi-permissive temperature 30ºC. Recombinant Lys + colonies were selected in 2% galactose SC medium without lysine. Recombination frequencies were calculated as the median value of six independent colonies.

## Results

### The Sμ350 sequence triggers R-loop formation in yeast genes

**Figure 1.**
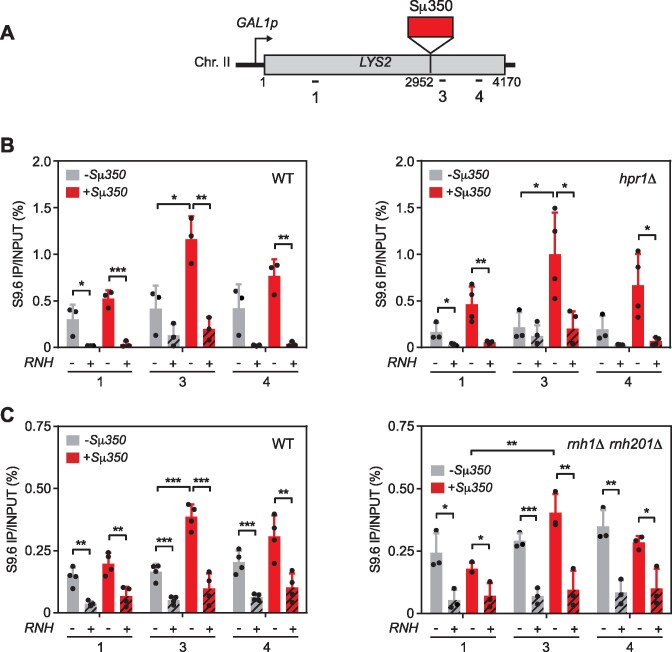
Analysis of R-loops at the Sμ350 sequences. (**A**) Schematic representation of the *GAL1p-LYS2* system in the chromosome II of *S. cerevisiae*, with or without Sμ350 sequence inserted at position 2952 of the *LYS2* gene. Relative position of the qPCR primers is shown. (**B**) DRIP-qPCR using the S9.6 antibody in wild-type (GLY and GLSd) and *hpr1Δ* mutant (GLYH and GLSdH) in different regions of *LYS2* with (+Sμ350) or without Sμ350 (-Sμ350) either non-treated (RNH-) or treated (RNH+) *in vitro* with RNase H after 16h of transcription induction. (**C**) DRIP-qPCR with the S9.6 antibody in wild-type (GLY and GLSd) and *rnh1Δ rnh201Δ* double mutant (GLYRH and GLSdRH) in the same regions and conditions as in B. Mean and SD of ≥3 samples are plotted for (B) and (C). *P ≤ 0.05; **P ≤ 0.01; ***P ≤ 0.001 (two-tailed Student's *t*-test).

To address how transcription progresses through R-loop-forming regions, we inserted the murine R-loop-prone GC-rich sequence Sμ350 inside the endogenous *LYS2* gene of *S. cerevisiae*. Sμ350 is a 350 bp DNA sequence formed by 15 tandem repeats of the Sμ consensus sequence from the murine immunoglobulin heavy chain, previously shown to be R-loop prone (27,34,48). We used CRISPR/Cas9 technology to insert it at position 2942 bp of the *LYS2* gene, from which we previously replaced its promoter by the galactose-inducible *GAL1* promoter (*GAL1::LYS2* construct) (Supplementary Figure S1A), to generate the reporter gene *GAL1::lys2::Sμ350* (Figure 1A). Next, we measured R-loops by DNA-RNA hybrid immunoprecipitation (DRIP) with the S9.6 antibody in both systems. Insertion of Sμ350 caused an increase in S9.6 signal throughout the *lys2* copy that is only significantly different from the signal at *GAL1::LYS2* at the region where Sμ350 is inserted (region 3, Figure 1B). *In vitro* treatment of the samples with RNase H, which degrades the RNA moiety of DNA-RNA hybrids, eliminates the signal validating the specificity of the S9.6 antibody for hybrids (Figure 1B).

If Sμ350 hybrids are similar in nature to hybrids formed in natural yeast DNA sequences and, therefore, controlled by the same factors, we wondered whether lack of such factors increased R-loops at the Sμ350 fragment inserted. Thus, we assessed the impact of the *hpr1Δ* mutant of the THO complex (14), and mutants of the RNases H1 and H2 with redundant functions, resolving DNA-RNA hybrids (49) on the Sμ350 DNA–RNA hybrids. Unexpectedly, the frequency of R-loops in *hpr1Δ* and *rnh1Δ rnh201Δ* mutants remained similar to that of wild-type strains (Figure 1B and C). We observed that *rnh1 rnh201* mutations increased R-loops throughout the *LYS2* gene in the system without Sμ350 (*GAL1::LYS2*), decreasing the difference with the Sμ350 reporter (*GAL1::lys2::Sμ350*) (Figure 1C). None of the mutations induced R-loops in a non-transcribed intergenic region of chromosome V (Supplementary Figure S1B and S1C). Therefore, insertion of the R-loop-prone GC-rich sequence Sμ350 specifically increased R-loop formation in the *LYS2* gene. From now on, we will refer to both systems as +Sμ350 and –Sμ350 indicating whether they had Sμ350 inserted or not.

### Transcription elongation is delayed by R-loops

**Figure 2.**
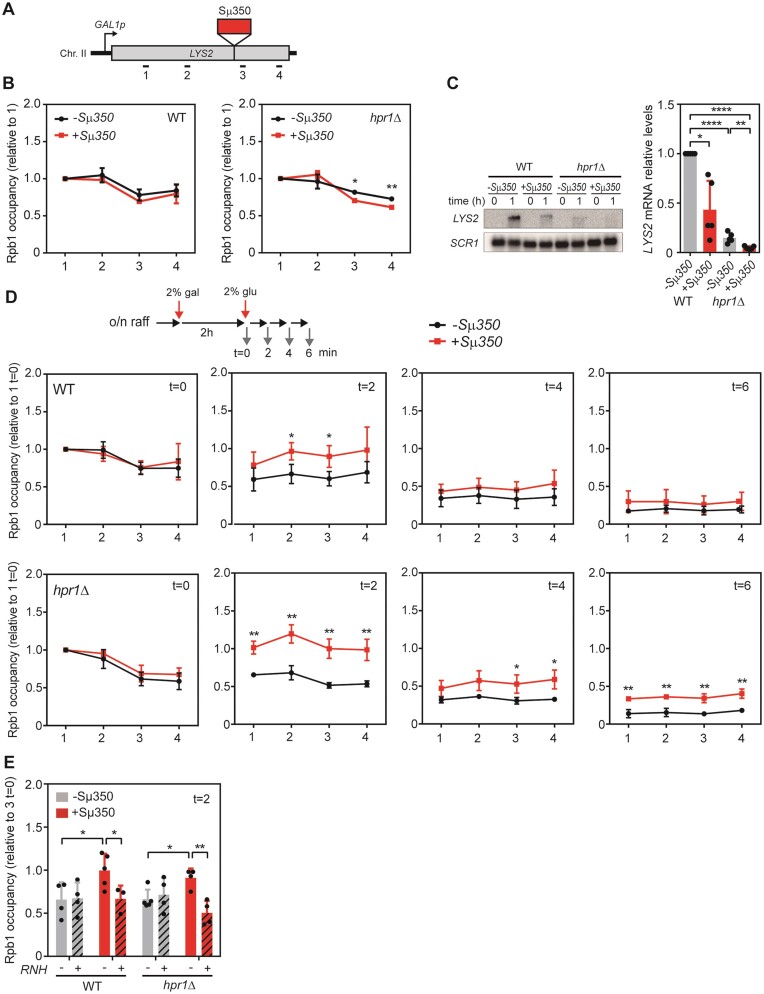
Effect of R-loop accumulation on RNAPII transcription. (**A**) Schematic representation of the *GAL1p-LYS2* system with or without the Sμ350 sequence inserted and the qPCR amplicons indicated. (**B**) Rpb1 ChIP-qPCR in different regions of *LYS2* gene (1, 2, 3 and 4) in wild-type (GLY and GLSd) or *hpr1Δ* cells (GLYH and GLSdH) with (+Sμ350) or without Sμ350 (−Sμ350) during steady-state transcription. (**C**) Northern blot assay of *LYS2* mRNA using the same strains as in B after 1 h of transcription induction. *SCR1* mRNA was used as loading control. (**D**) Rpb1 ChIP-qPCR in the same regions and conditions as in B 2h after induction (t = 0) and 2, 4 and 6 minutes after transcription inhibition by glucose addition (*t* = 2, 4 and 6). (**E**) Rpb1 ChIP-qPCR as in D in region 3, using cells transformed with either pRS416 (RNH-, solid bars) or pGALRH1 (RNH+, hatched bars) after 2 min of transcription inhibition (*t* = 2). Mean and SD of ≥3 samples are plotted for (B), (C), (D) and (E). *P ≤ 0.05; **P ≤ 0.01; ***P ≤ 0.001; ****P ≤ 0.0001 (two-tailed Student's *t*-test or one sample *t*-test in B). In (A), Rpb1 levels are relative to Rpb1 in region 1 in each condition. In (B) *LYS2* RNA levels are relative to the levels in the WT strain without Sμ350 (GLY). In (C), Rpb1 levels are relative to Rpb1 in region 1 at *t* = 0 in each condition. In (D), Rpb1 levels are relative to Rpb1 in region 3 at *t* = 0 in each condition.

Once validated, we analyzed transcription in the +Sμ350 and –Sμ350 systems by monitoring RNAPII occupancy via chromatin immunoprecipitation (ChIP). In wild-type cells, we observed an almost identical distribution of RNAPII along both systems (Figure 2A and B) suggesting that RNAPII is not persistently stalled at the R-loops. This prompted us to examine the *hpr1* mutant. In agreement with previous results, transcription of the *lys2* sequence in the *hpr1Δ* mutant was reduced compare to the wild type (Figure 2C and Supplementary Figure S2) (50). However, comparison of RNAPII profiles in both systems, reveals a slight but significant decrease of RNAPII occupancy downstream of the Sμ350 insertion in the +Sμ350 system in the *hpr1Δ* mutant, not observed in the −Sμ350 system, consistent with a transcription elongation impairment caused by R-loops. Thus, the difficulty of RNAPII elongation through R-loop-prone sequences is observed in the absence of THO (Figure 2B). Consistently, northern blot analysis revealed a clear decrease of expression of the +Sμ350 system, both in wild-type and *hpr1Δ* mutant strains (Figure 2C).

Previous *in vivo* and *in vitro* studies support a negative role for R-loops in transcription elongation (24,25,28,29). Thus, although under steady state conditions we only detect a minor difference in RNAPII distribution between the +Sμ350 and -Sμ350 systems in *hpr1Δ* strains, we wondered whether transcription elongation was altered by R-loops. For this, we examined RNAPII elongation through *lys2* monitoring RNAPII by ChIP at 2, 4 and 6 min after transcription repression (Figure 2D). We clearly observed that 2 min after inhibition of transcription, the R-loop-prone system accumulates RNAPII with a statistically significant increase in the region where Sμ350 was inserted and upstream of it (region 2 and 3). This difference is lost at 4 and 6 min, when the majority of the RNAPIIs have terminated transcription. In the *hpr1Δ* mutant, the absence of the THO complex enhances the difference observed between the +Sμ350 and −Sμ350 systems, even at 4 and 6 min after switching off transcription (Figure 2D). *In vivo* RNase H overexpression reduces RNAPII levels in the R-loop-forming region (region 3 in the +Sμ350 system) in wild-type and mutant *hpr1* cells, indicating that RNAPII accumulation is R-loop dependent (Figure 2E).

### Rat1 removes RNAPIIs accumulating upstream of R-loops

**Figure 3.**
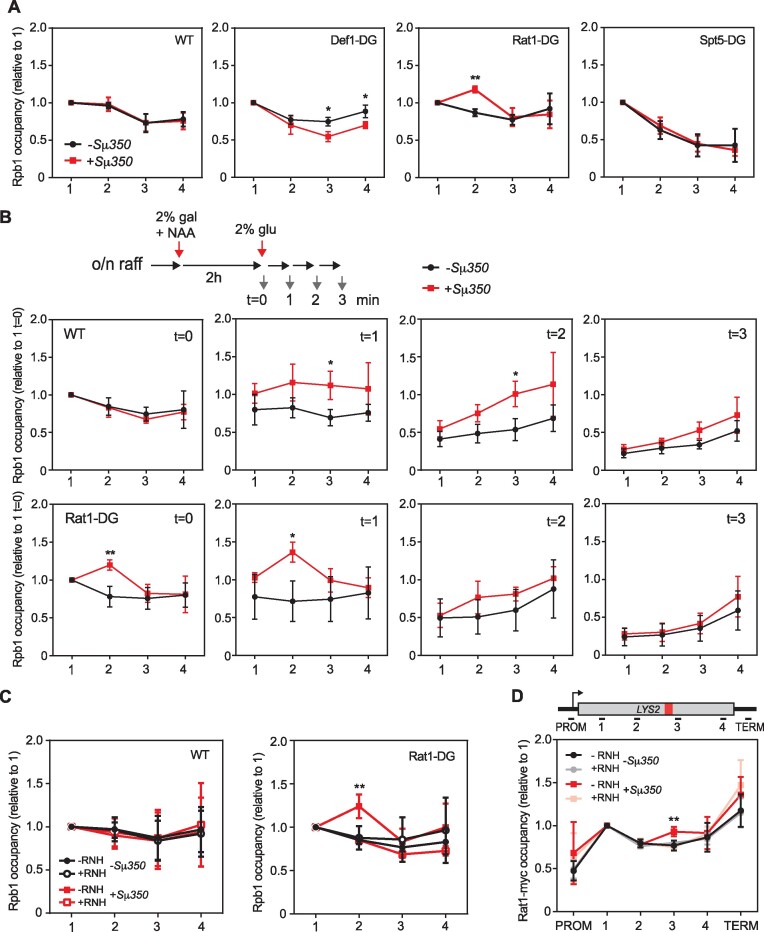
Effect of R-loop-accumulation on RNAPII upon depletion of Def1, Rat1 or Spt5. (**A**) Rpb1 ChIP-qPCR in the indicated regions (1, 2, 3 and 4) of *LYS2* gene with (+Sμ350) or without Sμ350 (–Sμ350) during steady-state transcription in wild-type (GLY and GLSd) conditions or after 2h of depletion of Def1 (GLYDF and GLSdDF), Rat1 (GLYRT and GLSdRT) or Spt5 (GLYSPT and GLSdSPT) by addition of 1mM NAA. (**B**) Rpb1 ChIP-qPCR in wild-type (GLY and GLSd) or upon depletion of Rat1 (GLYRT and GLSdRT) 2h after transcription induction (*t* = 0) or 1, 2 or 3 minutes after transcription inhibition by glucose addition (*t* = 1, 2 and 3). The experiment was performed after 2h of Rat1 depletion with 1 mM NAA. (**C**) Rpb1 ChIP-qPCR performed as in A in wild-type (GLY and GLSd) or Rat-DG strains (GLYRT and GLSdRT) transformed with either pRS413 (RNH–) or pRS413-GALRNH1 (RNH+). (**D**) Rat1-myc ChIP-qPCR using anti-myc antibody in Rat1 degron strain (RAT1-DG) strain without depleting Rat1 (GLYRT and GLSdRT). Mean and SD of ≥ 3 samples are plotted for **(**A), (B), (C) and (D). *P ≤ 0.05; **P ≤ 0.01 (two-tailed Student's *t*-test). In (A) and (C), Rpb1 levels are relative to Rpb1 in region 1 in each condition. In (B), Rpb1 levels are relative to Rpb1 in region 1 at *t* = 0 in each condition. In (**D**), Rat1 levels are relative to Rat1 levels in region 1 in each condition.

Since transcription elongation is lower in the presence of R-loops (Figure 2D), but no peaks of RNAPIIs are observed upstream of the Sμ350 region (Figure 2B), it is possible that a cellular mechanism operates to remove stalled RNAPIIs or restore transcription through the R-loop-forming sequence. So, we assayed whether stalled RNAPIIs could be eliminated either by degradation, similarly to when it is stalled at DNA damage, or by premature transcription termination. For this, we generated an auxin-inducible degron of Rat1, Spt5 and Def1 factors (RAT1-DG, Spt5-DG and DEF1-DG respectively) to disrupt transcription termination, elongation, or RNAPII degradation, respectively, and analyze whether any of these processes promote transcription through R-loops. We first confirmed that adding the AID tag does not alter RNAPII distribution along *lys2* alleles, as seen by ChIP in the absence of auxin (Supplementary Figure S3A), but allows the complete removal of these factors 90 min after the addition of auxin (Supplementary Figure S3B). Interestingly, in the absence of the transcription termination factor Rat1, we observed a peak of RNAPII upstream of the R-loop region, while depletion of Def1 or Spt5 either did not change or slightly reduced RNAPII levels at the +Sμ350 system (Figure 3A, Supplementary Figure S3C). Next, we analyzed RNAPII elongation by switching off transcription and monitoring RNAPII progression at short times, since most of the transcription was ended at times longer than 5 min. We confirmed that in wild-type cells, RNAPII proceeds more slowly through the template containing the R-loop-prone sequence, although 3 min after repressing initiation, most RNAPIIs had finished transcription in both templates. In the absence of Rat1, the accumulation of RNAPII observed after transcription inhibition upstream of Sμ350 increases, indicating that Rat1 is involved in facilitating the progression of RNAPII through this sequence (Figure 3B). However, as we previously observed for wild-type cells, 3 min after repressing *lys2* expression, most RNAPIIs have already finished transcription. These data indicate that Rat1 is involved in the release of RNAPII accumulated upstream of Sμ350. However, alternative mechanisms might still operate in its absence to either eliminate the RNAPIIs and/or the DNA–RNA hybrids.

To verify that the defect in RNAPII elongation observed upstream of the Sμ350 region is caused by R-loops, we overexpressed RNase H in cells depleted of Rat1. Under this condition, accumulation of RNAPII upstream of the Sμ350 sequence is suppressed, returning to the level of the −Sμ350 system (Figure 3C). Interestingly *lys2* expression after Rat1 depletion was also reduced in the +Sμ350 R-loop-prone template, similarly to wild-type cells. Although we observe a slight suppression upon RNase H overexpression, the lack of significant difference could be accounted for RNase H degrading the transcript that forms the DNA-RNA hybrid (Supplementary Figure S4). Therefore, DNA−RNA hybrids are a main cause of the RNAPII enrichment upstream of Sμ350 when Rat1 is depleted.

Next, we examined the location of Rat1 throughout the *LYS2* gene and we confirmed that Rat1 is accumulated at the gene body peaking at the termination region. Interestingly, Rat1 is enriched in the R-loop-forming system at the Sμ350 region in an RNase H sensitive manner, consistent with a role of Rat1 in elimination of RNAPIIs stalled at R-loops (Figure 3D).

**Figure 4.**
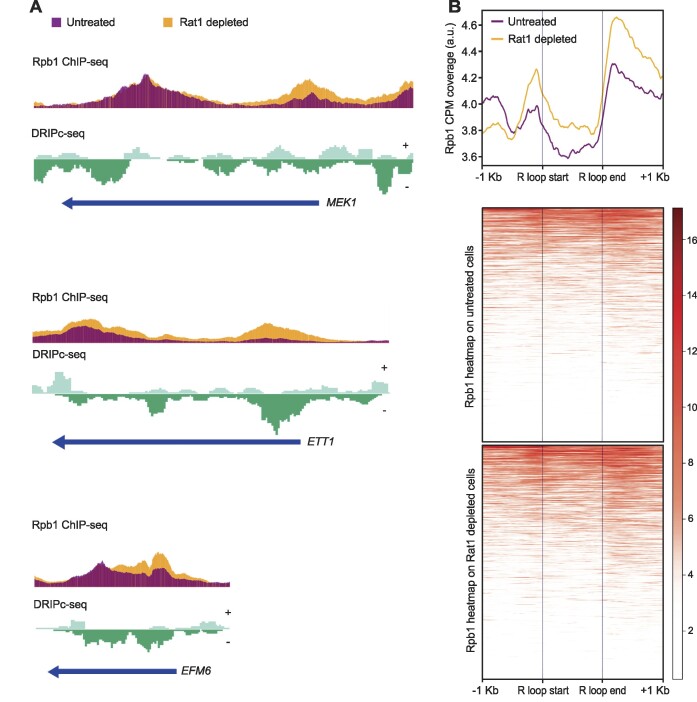
Rpb1 ChIP-seq profiles in Rat1-depleted cells at R-loop-forming regions identified by DRIPc-seq. (**A**) Representative screenshots of three genomic regions (*MEK1*, *ETT1*, and *EFM6*) showing the Rpb1 ChIP-seq signal in Rat1 depleted (yellow) or untreated (purple) conditions and DRIPc-seq signal mapped at Watson and Crick strands. (**B**) Upper panel: Metaplot analysis of Rpb1 ChIP-seq signals across untreated (purple) and Rat1-depleted (yellow) samples at DNA-RNA hybrid regions (±1 Kb) identified by DRIPc-seq. Lower panel: Heatmap displaying the distribution and intensity of Rpb1 signals at these regions, scaled from low (white) to high (red).

So far, we have analyzed the effect of depleting Rat1 exclusively on the transcription of the systems + or −Sμ350 that we generated. To expand the analysis to the whole genome, we used the published Rpb1 ChIP-seq data in a Rat1 anchor-away strain after depleting or not this factor from the nucleus (40) and compared RNAPII profile in naturally forming R-loop genomic regions previously determined in our group by DRIPc-seq (12). First, we defined the genomic coordinates of consistent R-loop-accumulating regions using chromstaR. Next, we analyzed Rpb1 mean coverage in these regions by deepTools’computeMatrix. Three representative genomic regions are shown (Figure 4A). We observed that removing Rat1 increases RNAPII occupancy in the body of the analyzed genes coinciding with R-loop rich regions. Metaplot analysis of Rpb1 at these regions reveals an increase of RNAPII in the absence of Rat1 (Figure 4B), supporting a role for this transcription termination factor in facilitating RNAPII progression through R loops at genomic scale. To assess whether Rat1 is present in R-loop-forming regions, we repeat the analysis using previously published Rat1 ChIP-seq data (40). We observed a peak of Rat1 downstream of R-loop-enriched regions (Supplementary Figure S5) supporting our previous result in the + Sμ350 system (Figure 3D). Altogether, these data suggest that Rat1 is recruited to the proximity of the RNAPIIs transiently paused at the R-loops and triggers premature transcription termination.

### RNase H action facilitates RNAPII elongation through R-loop-forming regions

**Figure 5.**
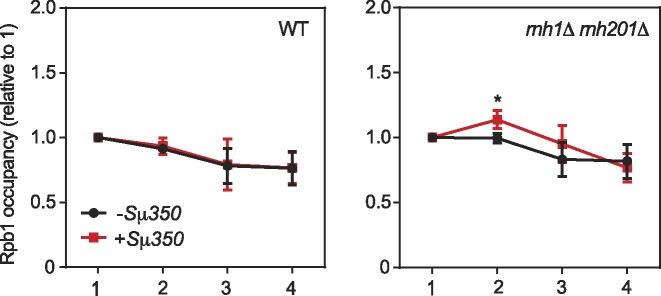
RNase H contribution to transcription of R-loop-prone sequences. Rpb1 ChIP-qPCR at the indicated regions (1, 2, 3 and 4) of *LYS2* gene with (+Sμ350) or without Sμ350 (−Sμ350) in wild-type (GLY and GLSd) or *rnh1Δ rnh201Δ* double mutant strains (GLYRH and GLSdRH) during steady-state transcription. Mean and SD of ≥3 samples are plotted. *P ≤ 0.05 (two-tailed Student's *t*-test). Rpb1 levels are relative to Rpb1 in region 1 in each condition.

As mentioned above, the fact that the accumulation of RNAPII detected upon Rat1 depletion is resolved 3 min after repressing transcription suggests that additional mechanisms operate to facilitate transcription through R-loop-prone regions. This prompted us to examine whether RNase H action could suppress RNAPII accumulation at R-loops. For this, we compared Rpb1 profile in the +Sμ350 and −Sμ350 systems in the double mutant *rnh1Δ rnh201Δ*. Interestingly, we detected that RNAPII accumulates slightly at and upstream of the R-loop-forming region exclusively in the +Sμ350 allele (regions 2 and 3) (Figure 5). R-loop level at the Sμ350 sequence was similar in the *rnh1Δ rnh201Δ* mutant and wild type (Figure 1C), ruling out an indirect effect caused by an increase in R-loops. The result suggests that RNase H facilitates elongation of RNAPII through R-loop-forming regions. Overall, the data indicate that transcription elongation is inhibited by R-loops present in the template, but this is rapidly counteracted by Rat1-mediated premature termination aided by RNase H.

### Rat1 depletion increases R-loop dependent genome instability

**Figure 6.**
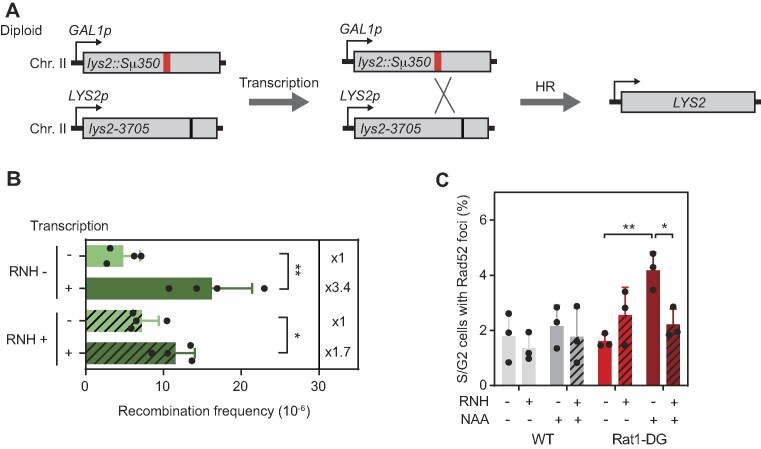
Genome instability induced by the R-loop-mediated RNAPII stalling. (**A**) Schematic representation of the recombination system in a diploid strain containing the *GAL1::lys2::Sμ350* and the *lys2-3705* alleles in each copy of chromosome II. Restoration of the *LYS2* gene occurs by repair with the homologous chromosome when damage is induced by the accumulation of R-loops in the *Sμ350* sequence. (**B**) Frequency of recombination in a wild-type strain (DGLSd) transformed with either pCM189-RNH1 (RNH+, solid bars) or pCM189 (RNH-, hatched bars) with or without transcription of the *lys2::Sμ350* allele. (**C**) Percentage of S/G2 cells with Rad52-YFP foci in WT (GLY) and Rat1-DG cells (GLYRT) transformed with pWJ1344 and either pRS313 (RNH-, solid bars) or pRS313-GALRNH1 (RNH+, hatched bars) plasmids. Cells were fixed after 3 h of 1mM NAA addition. Mean and SD of ≥3 samples are plotted for (B) and (C). *P ≤ 0.05; **P ≤ 0.01 (two-tailed Student's *t*-test).

RNAPII pausing or stalling can interfere with DNA replication and repair, leading to genome instability (1,51). Therefore, we wondered whether the delay in elongation imposed by Sμ350 was associated with DNA damage. To address this, we developed an allelic recombination system in a diploid strain carrying two versions of the *LYS2* gene in chromosome II: the *GAL1::lys2::Sμ350* allele and the point mutation allele *lys2-3705* at the homologous chromosome. Allelic recombination was determined by the frequency of Lys + recombinants selected in media without Lys (Figure 6A). We found first that transcription increased the frequency of spontaneous recombination 3.4 times, consistent with the known effect of transcription on recombination (52). RNase H overexpression did not alter the levels of transcription-associated recombination, likely due to a secondary effect increasing recombination (Figure 6B). However, if R-loop-stalled RNAPIIs are a source of genomic instability, it would be expected that Rat1 depletion would increase it. Since *RAT1* is an essential gene, we used the thermosensitive mutant *rat1-1* to assess recombination. For this, we generated by CRISPR/Cas9 the homozygous *rat1-1* diploid strain in the *GAL1::lys2::Sμ350/ lys2-3705* background. Although there is an increase in transcription-independent recombination in the *rat1-1* mutant, transcription of the *GAL1::lys2::Sμ350* gene did not significantly enhance recombination in the *rat1-1* diploid strain (Supplementary Figure S6). Since the *rat1-1* mutation has been shown to alter transcription elongation (53), it is possible that RNAPII do not accumulate at the Sμ350 sequence as previously observed in the Rat1-degron strain. To overcome this possible limitation, we assessed genome instability under the conditions in which RNAPII accumulation was detected, this is 2h after Rat1 depletion in the Rat1-degron strain. Genome instability was determined by the levels of Rad52 foci, a marker of recombinogenic double strand breaks (DSBs), as detected by immunofluorescence (IF) (47). We found that removal of Rat1 increased the percentage of cells with Rad52 foci 2.8 times. Importantly, this increase was RNase H sensitive (Figure 6C) indicating that Rat1 prevents R-loop-mediated DNA breaks. We conclude therefore that RNAPII elongation impairment at R-loop-forming templates, if not resolved by Rat1, significantly increase DNA breaks and genome instability, consistent with the conclusion that Rat1 promotes transcription through R-loops thus preventing genome instability.

## Discussion

In this study we show that R-loops delay RNAPII-mediated transcription elongation, temporarily pausing or stalling the polymerase. The transcription termination factor Rat1, possibly with the help of RNase H, is involved in releasing these RNAPIIs by premature transcription termination. If this transcription safety mechanism fails, DNA damage increases, and could ultimately lead to genome instability. In this context, Rat1 could be considered a surveillance factor that limits the accumulation of RNAPIIs at R-loop-prone genomic regions, thereby reducing the toxic effect of these structures on the cell.

Earlier *in vitro* and *in vivo* studies have clearly established that R-loops inhibit transcription elongation (14,23,24,26,27,29). However, it is not known whether it is the RNAP whose nascent RNA hybridizes with the DNA that is blocked, or whether it is the RNAP that collides with the hybrid. Experimental evidence supports both scenarios. In support of the first model, transcription of a template with a pre-formed R-loop reduces the elongation efficiency of the RNAPII (28,29,54), consistent with R-loops acting as roadblocks for the elongating polymerase. Unlike replication, where DNA polymerase is assisted by helicases to open DNA, during transcription, RNAPII unwinds the DNA double helix itself, extending the transcriptional bubble created at the promoter (55). Thus, RNAPII may find it difficult to pass through a triple helix structure such as R-loops without the aid of a helicase that unwinds at least the 5′ end of the DNA–RNA hybrid to remove it. An alternative but not incompatible scenario would be that the elongating RNAPII, whose transcript tethers back into the DNA forming the hybrid, is the one stalled. In this case, the resulting DNA-RNA hybrid prevents the RNAPII from rotating freely around the DNA helix, and the forces generated by the RNA wrapping around the DNA could culminate in the arrest of the RNAPII (30). Although we observed an RNAPII-accumulation upstream of the Sμ350 sequence (Figures 2 and 3), we could not exclude the presence of RNAPIIs attached to the DNA by the hybrid downstream of the R-loop, at a lower level. These downstream RNAPIIs and/or the R-loops formed, would contribute to blocking the incoming RNAPIIs, resulting in their accumulation detected in our system.

**Figure 7.**
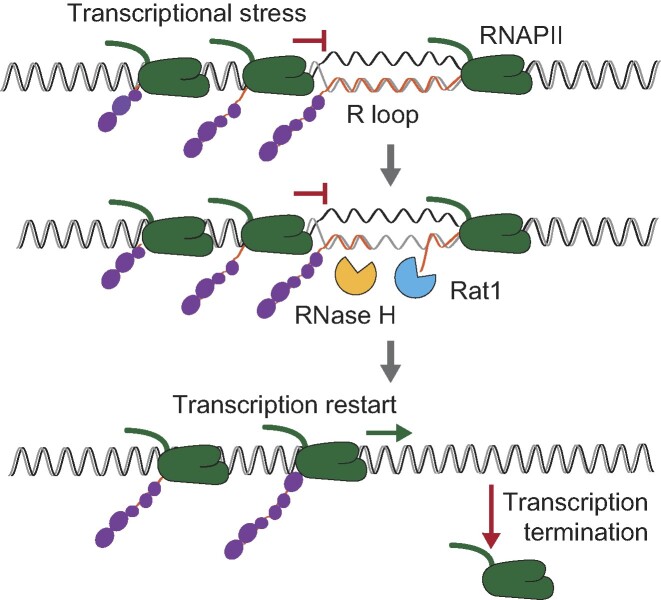
Suggested model: RNAPII is naturally stalled by R-loops during transcription. RNase H, by degrading the RNA motif of the DNA-RNA hybrid, creates an entry site for Rat1 that mediates premature termination and resumes the upcoming transcription.

Rat1/XRN2 in an essential 5′-3′ exonuclease that access the unprotected 5′ end generated when Ysh1/CPSF73 cleaves the nascent transcript at the polyadenylation signal, and triggers transcription termination upon reaching the RNAPII (56,57). Here we have found a novel role for Rat1 in removing RNAPIIs stalled by R-loops. An intriguing caveat is how Rat1 gains access to the RNA to release those RNAPIIs. One possibility is that the 5′ cap that protects the nascent RNA is eliminated. In support of this, mammalian XRN2 has been functionally and physically linked to the decapping factors Dcp1, Edc3 and Dcp2 (58). In yeast, although no physical interaction with Dcp1 has been reported, Rat1 interacts with Rai1, an mRNA endonuclease involved in decapping (59). Once uncapped, the nascent mRNA would be a substrate for Rat1/XRN2, as previously shown for certain viral and human genes whose expression is reduced by premature termination (58,60,61), or for aberrant transcripts that fail to form the 5′ cap correctly (59,62). Rat1/XRN2 might require the assistance of RNA helicases to unwind the DNA-RNA hybrid prior to degradation. Thus, DDX5 and SETX help XRN2 to terminate transcription at R-loop prone regions (22,63) while Sen1 facilitates Rat1 mediated transcription termination (64). Alternatively to decapping, an entry site for Rat1 could be created by degradation of the R-loop RNA motif by RNase H (Figure 7). Indeed, we observed that mutation of both RNases H causes RNAPII accumulation specifically in the R-loop enriched +Sμ350 system (Figure 5), suggesting that RNase H might be involved in facilitating RNAPII release at R-loop-prone regions. Previously published data show that RNA cleavage by RNase H triggers transcription termination (65–67), supporting a role for RNase H not only in removing DNA-RNA hybrids, but also in removing stalled RNAPIIs by helping Rat1/XRN2. In contrast to decapping, the coordinated action of RNase H and Rat1 would only disassemble an RNAPII sitting downstream of Sμ350, whose transcript forms the DNA–RNA hybrids. Nevertheless, removal of the hybrid and the stalled RNAPII will clear the pathway for the incoming RNAPIIs (Figure 7). Regardless of the mechanism of access, Rat1 would degrade the transcript, resulting in the reduction of the lys2::Sμ350 mRNA that we observed (Figure 2C).

Although our data clearly indicate that Rat1 is involved in the premature termination of RNAPIIs encountering R-loops, it is unlikely to be the only activity responsible for this task. Removal of Rat1 from the cell delays RNAPII elongation in the Sμ350 system, causing RNAPII to stall upstream of the R-loop-prone sequence. However, the RNAPII accumulation disappears a few minutes after transcription repression (Figure 3B). This could be the result of RNase H or RNA helicases removing the DNA-RNA hybrid, although it is unclear whether an RNAPII would restore elongation once the R-loop is removed or whether other factors might be required. For instance, RNAPII may need the help of the elongation factor TFIIS if the R-loop induces RNAPII backtracking. If this is the case, it could create a positive feedback loop, as backtracked RNAPIIs promote the formation of R-loops in front of them, which would block transcription (31). Another alternative factor that could be eliminating R-loop-stalled RNAPII is Sen1/SETX. In the absence of Rat1/XRN2, if R-loop-stalled RNAPIIs are not removed before S phase, they could encounter the replisome and cause transcription replication conflicts. The helicase Sen1/SETX associates with the replisome and helps it to replicate RNAPII transcribed regions (68,69). In yeast, Sen1 has recently been implicated in the release of RNAPIIs that cause transcription-replication conflicts through a transcription termination independent mechanism (20,70,71) and in removing R-loops during S phase (12). Thus, Sen1 might contribute to the elimination of those R-loop-blocked RNAPIIs at transcription replication conflicts during S phase, but it may not be a major actor at G1, when Rat1 may be the key player.

Rat1 mediated premature termination of RNAPII stalled by R-loops could play an important role in gene expression regulation for the cell if it occurs at the 5′ region of genes. The restrictions that RNA encounter to tether back to DNA are overcome at the 5′ region of genes where the nascent transcript is still short enough to invade the DNA. Indeed, under natural situations R-loops appear predominantly in the vicinity of promoters (20,72). In this case, RNAPIIs would pause near to the promoters, a situation indistinguishable from promoter-proximal pausing, and XRN2-mediated premature termination would reduce gene expression. Experimental evidence suggests that this is indeed the case, as XRN2 depletion increases RNAPII occupancy in the body of genes (72).

Another context where Rat1 mediated RNAPII release could have biological relevance is DNA repair. DNA breakage facilitates DNA-RNA hybrid formation (73) and these hybrids might prevent its repair (33). It would be interesting to know whether the RNAPII would remain attached to these DNA-RNA hybrids and whether Rat1 would be needed to remove it in order to facilitate the repair of DSBs. In support of this idea, after cell irradiation, XRN2 colocalizes with DSBs and R-loops and if XRN2 is depleted from cells, DNA repair is delayed (74).

Taken together, our data supports the model in which Rat1 triggers premature transcription termination of RNAPIIs stalled by R-loops. By these means Rat1 facilitates transcription at R-loop prone regions. A remaining question is whether Rat1 could facilitate the transcription of other types of intrinsically difficult to transcribe genomic regions or other DNA templated processes like DNA repair.

## Supplementary Material

gkae033_Supplemental_File

## Data Availability

The data underlying this article are available in the article and in its online supplementary material.

## Notes

Present address: José Antonio Mérida-Cerro, CIMA Universidad de Navarra, Pamplona, Spain.
