# Supplementary material for: Rat1 promotes premature transcription termination at R-loops
Source: Nucleic Acids Res. 2024 Jan 28;52(7):3623–35. doi: 10.1093/nar/gkae033 (PMC11039981; doi:10.1093/nar/gkae033)
Supplement: gkae033_Supplemental_File [file gkae033_supplemental_file.pdf]

Supplementary Table 1. Yeast strains used in this study.

**Strain**	**Genotype**	**Source**
YBP249	*MATa ade2-1 can1-100 his3-11,15 leu2-3,112 trp1-1 ura3-1 bar1Δ RAD5*	75
YBP250	*MATα ade2-1 can1-100 his3-11,15 leu2-3,112 trp1-1 ura3-1 bar1Δ RAD5*	75
YMK612	*MATa leu2-3,112 URA3-1::ADH1-AtTIR1-9Myc trp1-1 can1-100 ura3-1 ade2-1 his3-11,15*	37
RATDG	*MATa leu2-3,112 trp1-1 can1-100 ura3-1 ade2-1 his3-11,15 URA3-1::ADH1-AtTIR1-9Myc (URA3) rat1::rat1-aid (hphB)*	This study
SPTDG	*MATa leu2-3,112 trp1-1 can1-100 ura3-1 ade2-1 his3-11,15 URA3-1::ADH1-AtTIR1-9Myc (URA3) spt5::spt5-aid (hphB)*	This study
HPBAR1-R	*MATa leu2-3,112 trp1-1 can1-100 ura3-1 ade2-1 his3-11,15 bar1Δ hpr1ΔHIS3* RAD5	12
GLY	*MATa trp1-1 ura3-1 ade2-1 his3-11,15 can1-100 leu2-3,112 NATNT2::GAL-LYS2 bar1Δ RAD5*	33
GLY-B	*MATα trp1-1 ura3-1 ade2-1 his3-11,15 can1-100 leu2-3,112 NATNT2::GAL-LYS2 bar1Δ RAD5*	33
GLYDG	*MATa trp1-1 URA3-1::ADH1-AtTIR1-9Myc ade2-1 his3-11,15 can1-100 leu2-3,112 NATNT2::GAL-LYS2 bar1Δ RAD5*	This study
GLYH	*MATa trp1-1 ura3-1 ade2-1 his3-11,15 can1-100 leu2-3,112 hpr1::HIS3 NATNT2::GAL-LYS2 bar1Δ RAD5*	This study
GLYRH	*MATa trp1-1 ura3-1 ade2-1 his3-11,15 can1-100 leu2-3,112 rnh1::KanMX6 rnh201::HygMX6 NATNT2::GAL-LYS2 bar1Δ RAD5*	This study
GLYRT	*MATa trp1-1 URA3-1::ADH1-AtTIR1-9Myc ade2-1 his3-11,15 can1-100 leu2-3,112 NATNT2::GAL-LYS2 rat1::rat1-aid (hphB) bar1Δ RAD5*	This study
GLYSPT	*MATa trp1-1 URA3-1::ADH1-AtTIR1-9Myc ade2-1 his3-11,15 can1-100 leu2-3,112 NATNT2::GAL-LYS2 spt5::spt5-aid (hphB) bar1Δ RAD5*	This study
GLYDF	*MATa trp1-1 URA3-1::ADH1-AtTIR1-9Myc ade2-1 his3-11,15 can1-100 leu2-3,112 NATNT2::GAL-LYS2 def1::def1-aid (hphB) bar1Δ RAD5*	This study
GLSd	*MATa trp1-1 ura3-1 ade2-1 his3-11,15 can1-100 leu2-3,112 NATNT2::GAL-LYS2-Sμ350 bar1Δ RAD5*	This study
GLSd-B	*MATα trp1-1 ura3-1 ade2-1 his3-11,15 can1-100 leu2-3,112 NATNT2::GAL-LYS2-Sμ350 bar1Δ RAD5*	This study

GLSdDG	*MATα trp1-1 URA3-1::ADH1-AtTIR1-9Myc ade2-1 his3-11,15 can1-100 leu2-3,112 NATNT2::GAL-LYS2-Sμ350 bar1Δ RAD5*	This study
GLSdH	*MATa trp1-1 ura3-1 ade2-1 his3-11,15 can1-100 leu2-3,112 hpr1::HIS3 NATNT2::GAL-LYS2-Sμ350 bar1Δ RAD5*	This study
GLSdRH	*MATa trp1-1 ura3-1 ade2-1 his3-11,15 can1-100 leu2-3,112 rnh1::KanMX6 rnh201::HygMX6 NATNT2::GAL-LYS2-Sμ350 bar1Δ RAD5*	This study
GLSdRT	*MATa trp1-1 URA3-1::ADH1-AtTIR1-9Myc ade2-1 his3-11,15 can1-100 leu2-3,112 NATNT2::GAL-LYS2-Sμ350 rat1::rat1-aid (hphB) bar1Δ RAD5*	This study
GLSdSPT	*MATa trp1-1 URA3-1::ADH1-AtTIR1-9Myc ade2-1 his3-11,15 can1-100 leu2-3,112 NATNT2::GAL-LYS2-Sμ350 spt5::spt5-aid (hphB) bar1Δ RAD5*	This study
GLSdDF	*MATa trp1-1 URA3-1::ADH1-AtTIR1-9Myc ade2-1 his3-11,15 can1-100 leu2-3,112 NATNT2::GAL-LYS2-Sμ350 def1::def1-aid (hphB) bar1Δ RAD5*	This study
YLYS2	*Matα trp1-1 ura3-1 ade2-1 his3-11,15 can1-100 leu2-3,112 lys2-3705 bar1Δ RAD5*	33
YLR2	*Matα trp1-1 ura3-1 ade2-1 his3-11,15 can1-100 leu2-3,112 lys2-3705 rat1-1 bar1Δ RAD5*	This study
GLSR	*MATa trp1-1 ura3-1 ade2-1 his3-11,15 can1-100 leu2-3,112 NATNT2::GAL-LYS2-Sμ350 rat1-1 bar1Δ RAD5*	This study
DGLSd	*MATa/α trp1-1/ trp1-1 ura3-1/ ura3-1 ade2-1/ ade2-1 his3-11,15/ his3-11,15 can1-100/ can1-100 leu2-3,112/ leu2-3,112 lys2-3705/NATNT2::GAL-LYS2-Sμ350 bar1Δ/ bar1Δ RAD5/RAD5*	This study
DGLSdRT	*MATa/α trp1-1/ trp1-1 ade2-1/ ade2-1 his3-11,15/ his3-11,15 can1-100/ can1-100 leu2-3,112/ leu2-3,112 lys2-3705/NATNT2::GAL-LYS2-Sμ350 rat1-1/ rat1-1 bar1Δ/ bar1Δ RAD5/RAD5*	This study

Supplementary Table 2. Plasmids used in this study.

**Name**	**Description**	**Source**
pRS313	YCp vector with *GAL1* promoter and *HIS3* marker.	76
pRS313-GALRNH1	pRS313 containing the *GALp::RNH1* fusion.	77
pRS416	YCp vector with *GAL1* promoter and *URA3* marker.	76
pGALRH1	pRS416 plasmid containing the *GAL1p::RNH1* fusion.	R.J. Crouch
pCM189	Yeast centrosomal expression vector driven by the tetO7-CYC1 promoter.	78
pCM189-RNH1	RNH1 under tetO7-CYC1 control in the pCM189 plasmid.	79
pFA6-NATnt2-GALp	pFA6a plasmid containing the NatNT2-GAL1p cassette.	J. LaFuente-Barquero
pML104	pRS426 plasmid expressing Cas9 and containing a guide RNA expression cassette with a BclI-SwaI cloning site.	80
pML104-LYS2A	pML104 plasmid with a gRNA expression cassette targeting the position 2953 of the *LYS2* ORF.	33
pML104-3'mut	pML104 plasmid with a gRNA expression cassette targeting the position 3705 of the *LYS2* ORF to introduce one single nt deletion.	33
pRS413-SF	pRS413 plasmid containing Sμ350 from murine.	34
pFA6a-kanMX6	pFA6a plasmid containing the KanMX6 cassette.	35
pFA6a–hphNT1	pFA6a plasmid containing the HphNT1 cassette.	32
pFA6a-3HA-kanMX6	pFA6a plasmid containing a cassette with 3 copies of influenzavirus hemagglutinin (HA) epitope and KanMX6 selectable marker.	35
pHyg-AID-9myc	pSM409 plasmid containing AID-9myc tag with the selection marker hphNT1.	36
pWJ1344	YCp plasmid containing the Rad52::YFP fusion and *LEU2* marker.	46

Supplementary Table 3. Primers used in this study.

Name	Sequence (5' to 3')	Use
GAL1 PROM fw	GTTGTAATCGAGCTCGAATTCATCGATG	qPCR
GAL1 PROM rv	CGGAGGAGAGTCTTCCTTCGGAG	qPCR
LYS2 1 fw	GTGTGGATTTGATGGTATGTGTGA	qPCR
LYS2 1 rv	GCAGGGTCGATAACTGAAAAGG	qPCR
LYS2 2 fw	CTGGTTAGGTCCAAGAGATAGATTGT	qPCR
LYS2 2 rv	CAGTCACCGTTTGGTAGATAACGA	qPCR
LYS2 3 fw	CAGGGCCAAGGATGAAGAAG	qPCR
LYS2 3 rv	GTACCATAGGTGATACCTGCCTTT	qPCR
LYS2 4 fw	GCTCCGGAACTAGACGATAGGA	qPCR
LYS2 4 rv	CTGTCCATGCGGTGTCTTTCT	qPCR
LYS2 term fw	GGTTGAGCATTACGTATGATATGTCCA	qPCR
LYS2 term rv	CACCCGAAAAGAAGCTAAGTCTTTCT	qPCR
V1 up	TGTTCCTTTAAGAGGTGATGGTGAT	qPCR
V1 down	GTGCGCAGTACTTGTGAAAACC	qPCR
NAT-GALp LYS2 fw	GGCATCGCACAGTTTTAGCGAGGAAAACTCTTCAATAGTTTTGCCA GCGGGACATGGAGGCCCAGAATAC	genome editing
NAT-GALp LYS2 rv	GAAAGAGTTGGATTATCCAACTTCTCTATCCAGACCTTTTCGTTAGTCA TCTCCTTGACGTTAAAGTATAGAGG	genome editing
Sm350 LYS2 fw	GAAAAACAACAATTAATGTGTTTGTTACCGGTGTCACAGGATTTCTGG GCTCATTCCTGCAGCCCTGAGCTG	genome editing
Sm350 LYS2 rv	CTTGGCCCTGACGTGGGCAAACACTTTGAAACTGTAGTTCTTTGGAGA A CCTGCAGCCCGGGGGAT	genome editing
RAT1-AID fw	CTCGGAATAACAAGCAAAGTCGGTATGACAATTCAAGAGCAA ATAGGCGTCGTACGCTGCAGGTCGAC	genome editing
RAT1-AID rv	GATTTTATAAATTTGCGAAAACCTAAATTTACCATAAAATAAAAT GCGCATCGATGAATTCGAGCTCG	genome editing
DEF1-AID fw	ATGGCTACAACGGTTATGATTACAATTCTAAAAATTCAAGAGGTTTCTA C	genome editing
DEF1-AID rv	TCATTGTATTTTATTCCCCATTTCGTTTTTTATGTGGGAGGTTCTACTTC	genome editing
SPT5-AID up	GAAATAAGTCAAACTATGGTGGTAACAGTACATGGGGAGGTCATCGTA C GCTGCAGGTCGAC	genome editing
SPT5-AID dw	TTGATTTCTTCTTGGGTGATATTGGTTCTCCTTTTGGTGACATCGATGA A TTCGAGCTCG	genome editing
RNH1-MX6 fw	TTTAAATTAGTTAAAGTGTCACTCCTTGCTTATCGAAGGAACTATCGAT TCC TAATTATGGGATCCCCGGGTTAATTAAGG	genome editing
RNH1-MX6 rv	ATATATTTCTATTACAGGTACAACAGGTCCAGTAAGAAGCCAAGCAAAA AA CAGCATTATTAGTGGATCTGATATCATCG	genome editing
RNH201-Hyg fw	ATGAGAGTGTCGAAAAACCTTGAAAACAACTACTGCACACCAAATTGA TACGATTAAATGGGATCCCCGGGTTAATTAAGG	genome editing
RNH201-Hyg rv	TGAAGTTATGACATATGTAGTATTACATGAAGATATATAGTATGTGCAA ACTGGAGGTGAGAATTCGAGCTCGTTTAAAC	genome editing
LYS2 gRNA A	GATCTACATCCTTGCAGATTTGTTGTTTTAGAGCTAG	gRNA
LYS2 gRNA B	CTAGCTCTAAAACAACAAATCTGCAAGGATGTA	gRNA
LYS2 gRNA C	GATCGCCAATTCATTTTCTTTGGGGTTTTAGAGCTAG	gRNA

LYS2 gRNA D	CTAGCTCTAAAACCCCAAAGAAAATGAATTGGC	gRNA
LYS2 probe A fw	GCTACATATTCGTTACAGCTACCTCAGC	Probe
LYS2 probe A rv	GATGGATCGCTTAGCGCAGCAGTC	Probe
SCR1 .483 rv	GGCCACAATGTGCGAGTAAAT	Probe
SCR1 .99 dw	CCCAAAGGGCGTGCAAT	Probe
RAT1 gRNA-fw	GATCCTGTTGCCAGAGGAGCATAAGTTTTAGAGCTAG	gRNA
RAT1 gRNA-rv	CTAGCTCTAAAACTTATGCTCCTCTGGCAACAG	gRNA
Rat1-dplx-fw	CACAGCACAGTGAATGTCAGC	genome editing
Rat1-dplx-rv	CTAGCTCTAAAACTTATGCTCCTCTGGCAACAG	genome editing

Supplementary Table 4. Antibodies used in this study.

**Name**	**Use**	**Source**	**Identifier**
S9.6 antibody	DRIP (6 μg)	ATCC Hybri-doma cell line	Cat # HB-8730, RRID: CVCL_G144
c-Myc antibody [9E11] - ChIP Grade	ChIP (10 μg) Western (1:2000)	Abcam	Cat# ab56, RRID: AB_304976
Anti-beta Actin antibody	Western (1:1000)	Abcam	Cat# ab8227, RRID: AB_2305186
Purified anti-RNA Polymerase II RPB1 Antibody [8WG16]	ChIP (3 μg)	Biolegend	Cat# 61085, RRID: AB_2565554
Anti-Mouse IgG (whole molecule)-Peroxidase antibody produced in goat	Western (1:5000)	Sigma-Aldrich	Cat# A4416, RRID: AB_258167

REFERENCES

75. Moriel-Carretero M. and Aguilera A. (2010). A Postincision-Deficient TFIIH Causes Replication Fork Breakage and Uncovers Alternative Rad51- or Pol32-Mediated Restart Mechanisms. *Mol. Cell*,**37**, 690–701.

76. Sikorski R.S. and Hieter P. (1989) A system of shuttle vectors and yeast host strains designed for efficient manipulation of DNA in Saccharomyces cerevisiae. *Genetics* **122**, 19-27.

77. García-Benítez F., Gaillard H. and Aguilera A. (2017) Physical proximity of chromatin to nuclear pores prevents harmful R-loop accumulation

contributing to maintain genome stability. Proc *Natl Acad Sci USA* **114**, 10942-10947.

78. Garí E., Piedrafita L., Aldea M. and Herrero E. (1997) A set of vectors with a tetracycline-regulatable promoter system for modulated gene expression in Saccharomyces cerevisiae. *Yeast* **13**, 837-848.

79. Castellano-Pozo M., Santos-Pereira J.M., Rondón A.G., Barroso S., Andújar E., Pérez-Alegre M., García-Muse T. and Aguilera A. (2013) R-loops are linked to histone H3 S10 phosphorylation and chromatin condensation. *Mol Cell.* **52**, 583-590.

80. Laughery M.F., Hunter T., Brown A., Hoopes J., Ostbye T., Shumaker T. and Wyrick J.J. (2015) New vectors for simple and streamlined CRISPR-Cas9 genome editing in Saccharomyces cerevisiae. *Yeast* **32**, 711-20.

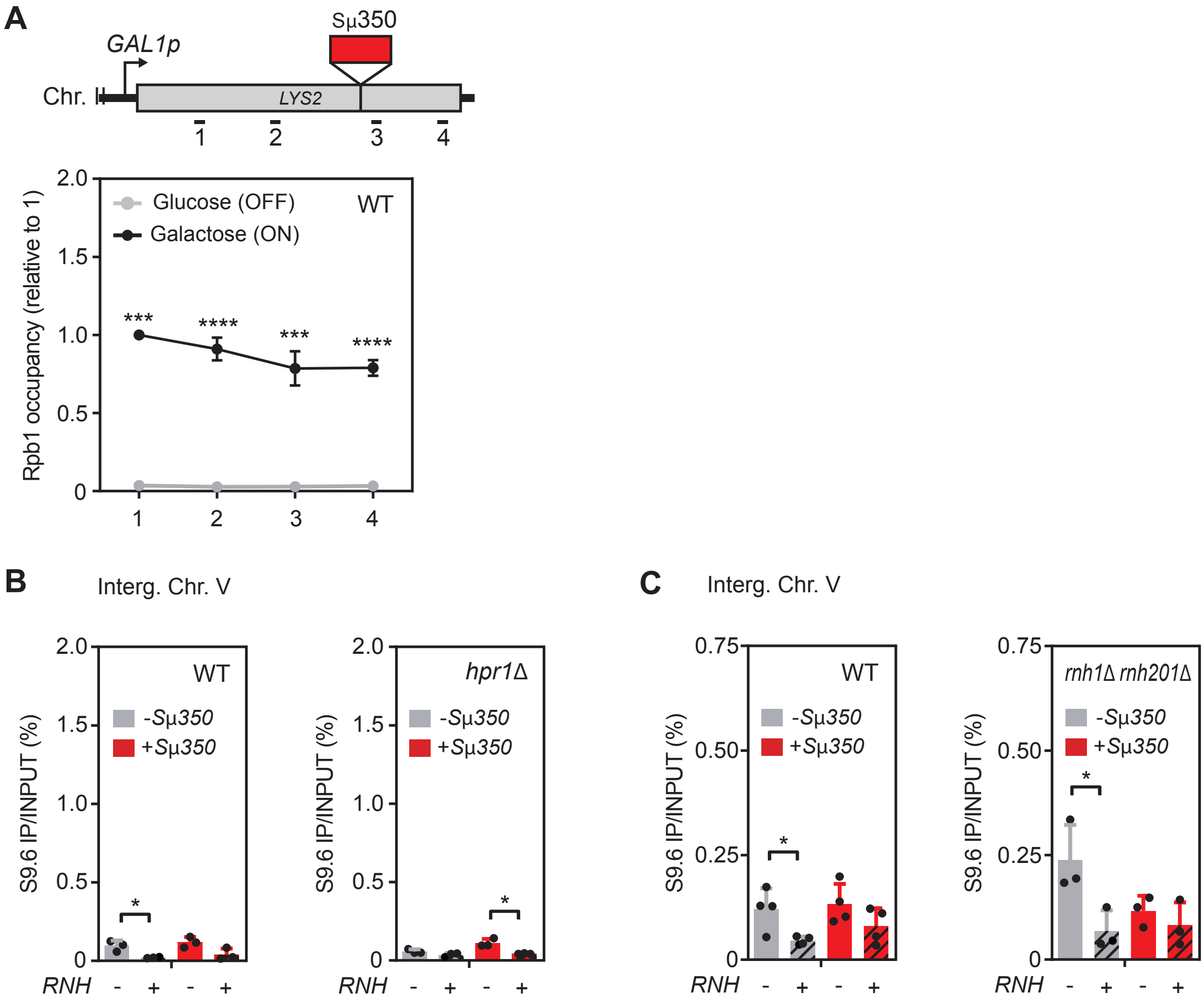

Figure S1. Analysis of the transcription induction of GAL1p-LYS2 system and R-loop formation specificity in the *GAL1p-LYS2:Sμ350* system. **(A)** Rpb1 ChIP-qPCR at the indicated regions of *LYS2* gene (1, 2, 3 and 4) in a wild-type strain (GLY) after 2h of transcription induction with galactose (ON) or inhibition with glucose (OFF). **(B)** DRIP -qPCR using the S9.6 antibody in a non-transcribed intergenic region of the chromosome V in wild-type (GLY and GLSd) or *hpr1*Δ mutant (GLYH and GLSdH) with (+Sμ350) or without Sμ350 (-Sμ350) either non-treated (RNH-) or treated (RNH+) *in vitro* with RNase H after 16h of transcription induction. **(C)** As in B, using wild-type (GLY and GLSd) or *rnh1*Δ *rnh201*Δ double mutant (GLYRH and GLSdRH) strains.

Mean and SD of ≥3 samples are plotted for (A), (B) and (C). *p≤0.05; ***p≤0.001; ****p≤0.0001 (two-tailed Student's t-test). In (A) Rpb1 levels are relative to Rpb1 in region 1 of galactose condition.

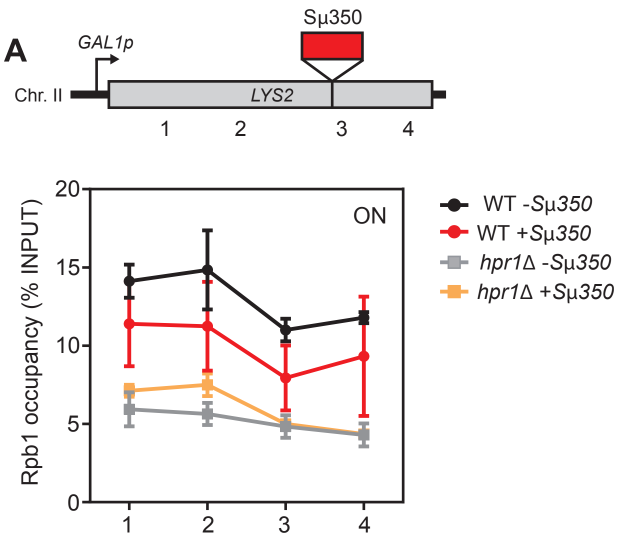

Figure S2. *hpr1* mutant reduces the global level of RNAPII. Rpb1 ChIP-qPCR in the *LYS2* gene with (+Sµ350) or without Sµ350 (-Sµ350) in wild-type (GLY and GLSd) or *hpr1*Δmutant (GLYH and GLSdH) background during steady-state transcription.

Mean and SD of ≥3 samples are plotted.

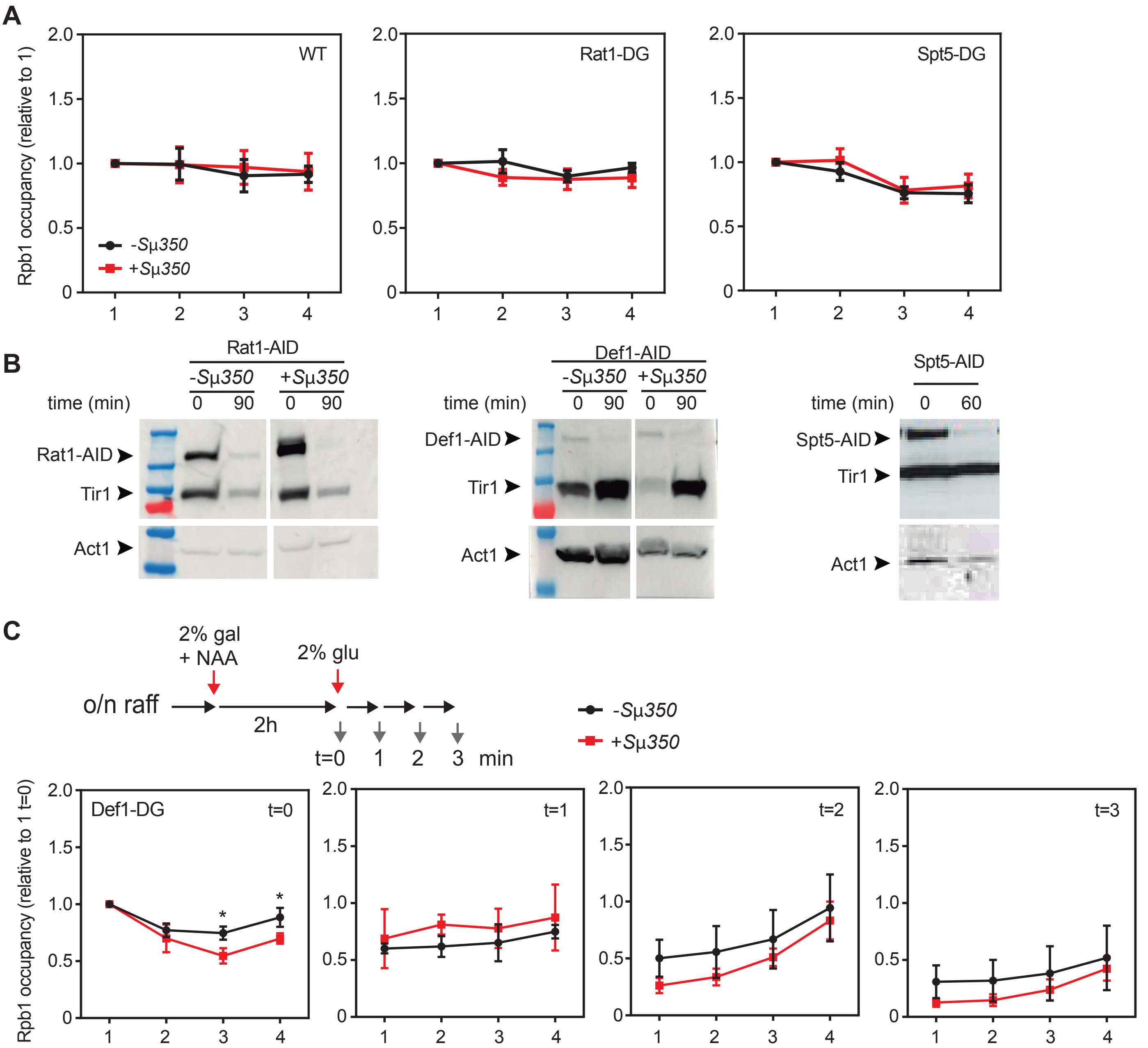

Figure S3. Depletion conditions for Rat1, Spt5 and Def1. **(A)** Rpb1 ChIP-qPCR at the indicated regions of the *LYS2* gene with (+Sμ350) or without Sμ350 (-Sμ350) in wild-type (GLY and GLSd), Rat1-DG (GLYRT and GLSdRT) and Spt5-DG (GLYSPT and GLSdSPT) strains without depleting these proteins (no NAA treatment). **(B)** Western blot assays using anti-myc antibody in Rat1-DG (GLYRT and GLSdRT), Def1-DG (GLYDF and GLSdDF) and Spt5-DG (SPTDG) strains before and after 60 or 90 min of 1mM NAA addition. **(C)** Rpb1 ChIP-qPCR in Def1-DG (GLYDF and GLSdDF) 2h after transcription induction (t=0) or 1, 2 or 3 minutes after transcription inhibition by glucose addition (t=1, 2 and 3). The experiment was performed after 2h of Def1 depletion with 1mM NAA.

Mean and SD of ≥3 samples are plotted for (A) and (C). *p≤0.05 (two-tailed Student's t-test). In (A), Rpb1 levels are relative to Rpb1 in region 1 in each condition. In (C), Rpb1 levels are relative to Rpb1 in region 1 at t=0 in each condition.

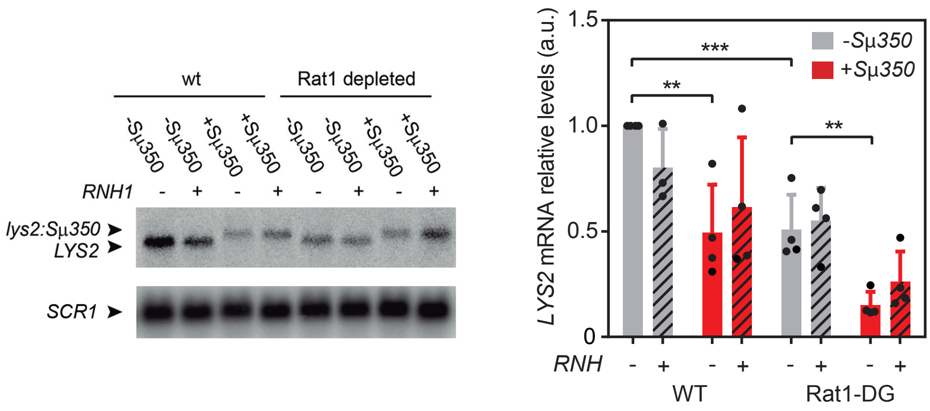

Figure S4. Effect of Rat1 depletion in *LYS2* mRNA levels. Northern blot assay of *LYS2* mRNA in wild-type (GLY and GLSd) or Rat-DG (GLYRT and GLSdRT) strains with (+Sμ350) or without Sμ350 (-Sμ350) after 1 h of transcription induction and 2h of Rat1 depletion using 1mM NAA. Cells were transformed with either pRS313 (RNH-) or pRS313-GALRNH1 (RNH+). *SCR1* mRNA was used as loading control.

Mean and SD of ≥3 samples are plotted. **p≤0.01; ***p≤0.001; (two-tailed Student's t-test). *LYS2* RNA levels are relative to the levels in the wild-type strain without Sμ350 (GLY) not overexpressing *RNH1*.

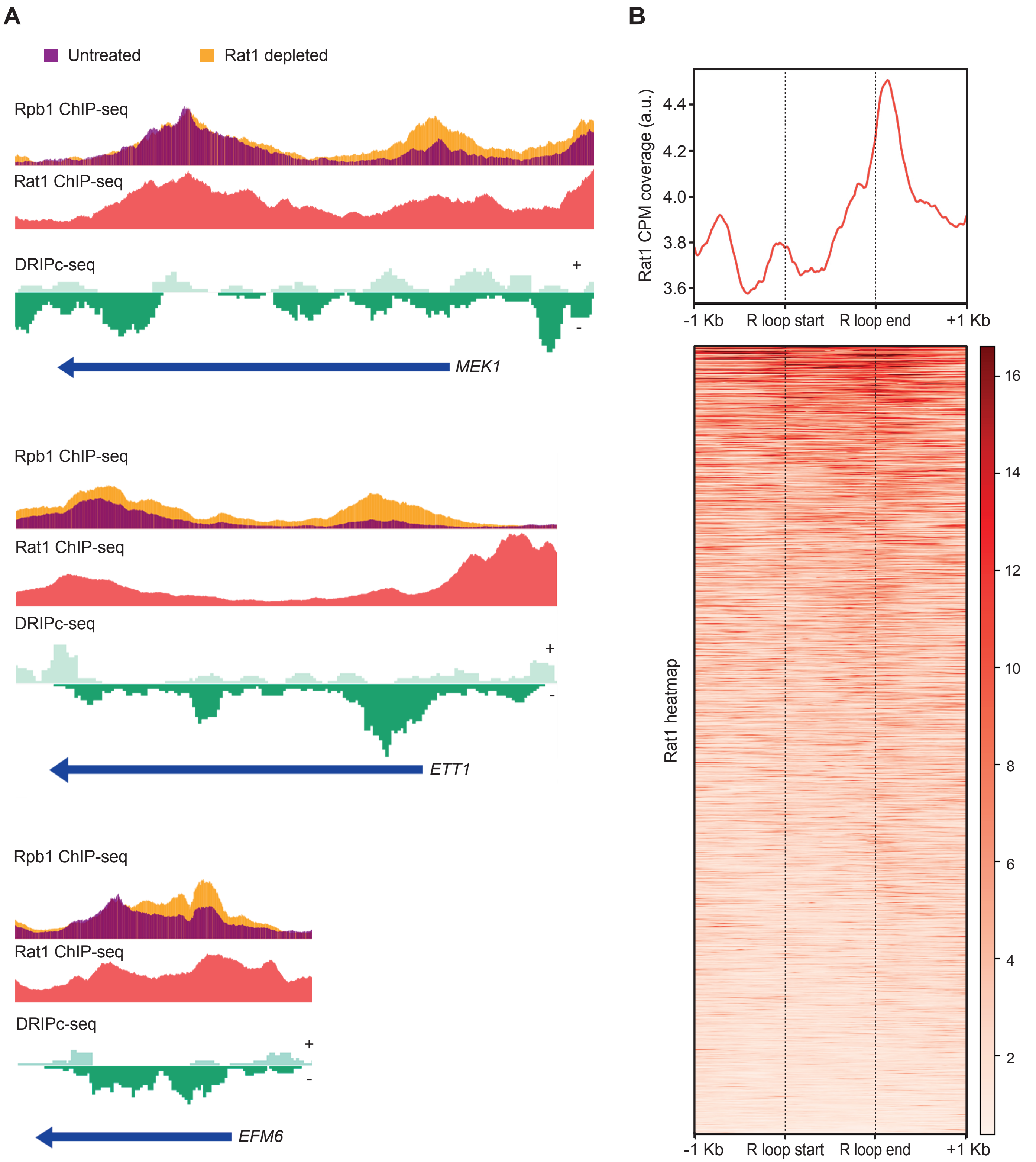

Figure S5. **(A)** Representative screenshots of *MEK1*, *ETT1* and *GOR1* genes showing the Rpb1 ChIP-seq signal in Rat1 depleted (yellow) or not (purple) conditions; Rat1 ChIP-seq signal and DRIPc-seq signal mapped at Watson and Crick strand. **(B)** Upper panel: Metaplot analysis of Rat1 ChIP-seq signals across DNA-RNA hybrid regions (±1 Kb) identified by DRIPc-seq. Lower panel: Heatmap displaying the distribution and intensity of Rat1 signals at these regions, scaled from low (white) to high (red).

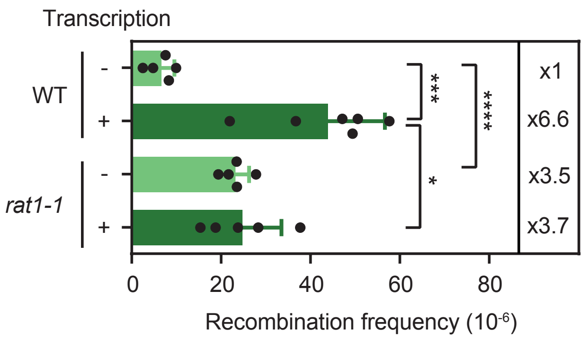

Figure S6. R-loop induced recombination in the diploid *rat1-1* mutant. Frequency of *lys2* recombination in wild-type (DGLSd) and *rat1-1* mutant (DGLSdRT) with or without transcription of the *lys2::Sμ350* allele.

Mean and SD of ≥5 samples are plotted. *p≤0.05; ***p≤0.001; ****p≤0.0001 (two-tailed Student's t-test).
